# Adherence to Preoperative Fasting Guidelines in Elective Surgical Patients

**DOI:** 10.7759/cureus.71554

**Published:** 2024-10-15

**Authors:** Abubakar I. Sidik, Alexandr Lishchuk, Alexander N Faybushevich, Aliu Moomin, Jonas Akambase, Vladislav Dontsov, Dmitriy Sobolev, Abdulmajid Ilyas Mohammad Shafii, Farjana Najneen, Gulten Ak, Derrar Ahlam, Maridia K Adam, Linus Baatiema, Charles Benneh, Paa Kofi T Adu-Gyamfi, Frank Agyapong, Kwesi Boadu Mensah

**Affiliations:** 1 Surgery, Rossiiskii Universitet Druzhby Narodov (RUDN) University, Moscow, RUS; 2 Cardiothoracic Surgery, A.A. Vishnevskiy Third Central Military Clinical Hospital, Moscow, RUS; 3 Cardiothoracic Surgery, Rossiiskii Universitet Druzhby Narodov (RUDN) University, Moscow, RUS; 4 Nutrition and Health, Rowett Institute, University of Aberdeen, Aberdeen, GBR; 5 Anatomy, Cairns Hospital, Cairns, AUS; 6 Cardiothoracic Surgery, Moscow Regional Research and Clinical Institute, Moscow, RUS; 7 Cardiology, European Medical Center, Moscow, RUS; 8 Cardiovascular Medicine, Rossiiskii Universitet Druzhby Narodov (RUDN) University, Moscow, RUS; 9 Health Sciences, Robert Gordon University, Aberdeen, GBR; 10 Health Services, Ghana Health Service, Wa, GHA; 11 Pharmacy and Pharmacy Practice, School of Pharmacy, Ulster University, Coleraine, GBR; 12 Nursing and Midwifery, Pentecost University College, Accra, GHA; 13 Pharmacology, College of Health Science, Kwame Nkrumah University of Science and Technology, Kumasi, GHA

**Keywords:** am patients, clear fluids, elective surgery, pm patients, preoperative fasting, solid food

## Abstract

Introduction: Preoperative fasting is recommended by international guidelines as a means to minimize the risk of aspiration of gastric content during induction of anesthesia or surgery. Prolonged preoperative fasting is, however, discouraged due to the associated side effects such as dehydration and electrolyte imbalance, which can negatively impact recovery after surgery. An initial quality improvement study revealed poor implementation of the best practice guidelines on preoperative fasting in three departments of a hospital and an institutional action plan was devised to enforce adherence to these guidelines. This present study aimed to assess compliance with the action plan and for that matter, adherence to international consensus on preoperative fasting in three surgical departments.

Methods: Adult patients undergoing elective cardiac, thoracic, and vascular surgery at a university teaching hospital were surveyed over four months (September October, November, and December of 2023). Data on the length of preoperative fasting was collected using a standardized questionnaire. A total of 306 patients who were scheduled for elective surgery were included in the study.

Results: Of the 306 patients, 139 (45.4%) had vascular surgeries, 108 (35.4%) received cardiac surgeries, and 59 (19.3%) had thoracic surgeries. For clear fluids, the overall median fasting time (Q1, Q3) was 4.5 (2.7, 7.4) hours, and for solid food, 14.5 (12.1, 19.0) hours. Extended abstinence from clear fluids and solid food for more than 12 hours was observed in 43 (14.1%) and 231 (75.5%) instances, respectively, while abstinence from solid food for more than 24 hours was noticed in 40 (13.1%) cases. When compared to patients having operations in the morning, those scheduled for afternoon surgery had longer median fasting periods from clear fluids and solid food, p<0.001: 6.2 (4.0, 12.0) hours vs. 3.4 (2.0, 5.2) hours for clear fluids and 16.7 (12.6, 22.6) hours vs. 13.2 (9.6, 15.2) hours for solid food, respectively.

Conclusion: Patients continue to abstain from clear fluids and solid food for extended periods of time, despite the fact that there is worldwide agreement regarding shorter periods of preoperative fasting. Compared to patients undergoing morning surgery, individuals hospitalized for afternoon procedures were more likely to fast for extended periods of time.

## Introduction

Preoperative fasting guidelines focus on “nil by mouth” and the use of medications to alter the volume and acidity of gastric contents before induction of anesthesia and surgery so as to minimize the risk of aspiration [1]. Patients traditionally fasted for over 12 hours until it was demonstrated in 1999 that shorter fasting time was not associated with a higher risk of adverse outcomes [2]. Although fasting from midnight has been easier for nursing staff to implement, it can cause hypoglycemia and fluid and electrolyte imbalance in patients. Moreover, shorter preoperative fasting times can be beneficial while ensuring minimal risk of aspiration during the induction of anesthesia [3,4].

The American Society of Anaesthesiologists guidelines recommend abstinence from clear fluids and solid food for two hours and four hours, respectively, since gastric clearance of these foods takes approximately the same amount of time [1,5]. Solid meals can be consumed until midnight and clear fluids can be consumed until 6:30 for patients on morning theatre lists (AM patients), as per the guidelines set forth by the Royal Infirmary of Edinburgh (RIE). Patients on afternoon lists (PM patients) should be allowed to have a modest breakfast before 7:00 and clear fluids till 11:00 [6]. The duration of preoperative fasting varies significantly among patients, with many experiencing prolonged periods without food before surgery [6-8]. Falconer et al. conducted an audit at a major UK center and found that patients refrained from consuming liquids and solid meals for a median (Q1, Q3) of approximately 9.4 (5.4, 12.8) hours and 13.5 (11.5, 16.0) hours, respectively [6].

Extended fasting is associated with adverse effects such as dehydration, which may lead to acute kidney injury, prolonged hospital stay, and poorer outcomes [9-11]. It also induces metabolic stress that causes depletion of the body’s glycogen stores, breakdown of muscle protein for gluconeogenesis, postoperative insulin resistance, and infective complications [12-14]. A study involving pediatric patients demonstrated that extended preoperative fasting contributes to poor hemodynamic conditions, such as low blood pressure, which complicates the induction of anesthesia [4]. Typically, information regarding fasting guidelines is communicated verbally and through written instructions during preoperative assessment clinics.

The primary objective of this study was to assess compliance with established standards by conducting a prospective audit of preoperative fasting durations among patients undergoing various elective surgical procedures.

## Materials and methods

Study design

A baseline audit was carried out in September 2022 in the surgical departments of a RUDN University-affiliated Hospital in Moscow to assess the extent to which best practice guidelines on preoperative fasting were followed. The baseline audit of 90 random patients found poor compliance in all departments. This action plan was developed to tackle guidelines nonadherence, and each department was entrusted with its implementation.

If a patient is scheduled for surgery in the morning, he/she must not have solid food after midnight but can have clear fluids until 06:30 am; if a patient is scheduled for surgery in the afternoon, he/she can have solid food until 07:00 am and clear fluids until 11:00 am; information on fasting should be conveyed to patients at pre-hospitalization and preoperative clinic visits; explanatory leaflets on fasting should also be provided to patients.

After the implementation of the action plan, a prospective follow-up re-audit was conducted for three months (October-December 2022) to evaluate compliance with the European Society of Anesthesiologists preoperative fasting guidelines.

Data collection

Patients were recruited preoperatively and were given a standardized questionnaire (supplementary information) as depicted in Table 4 of Appendices to obtain information on the length of preoperative fasting and the form of fasting instructions they received (written, verbal, or both). The total fasting duration (in hours) was measured from the start of fasting to the induction of anesthesia. The surgical approaches were minimally invasive, open, or conversion from a minimally invasive to an open technique. Patients’ demographics and hospitalization details were also collected.

According to the definition of surgical intervention by the National Confidential Enquiry into Patient Death and Outcome (NCEPOD), elective surgeries are those that are planned and scheduled prior to the patient's admission [15]. General anesthesia was used in 218 (71.2%) operations and regional anesthesia in some vascular procedures.

The inclusion criteria were adults (≥18 years) admitted for elective open surgery, including those scheduled for same-day admission and overnight stays; The criteria for exclusion included individuals admitted to the theatre following an acute care classification (NCEPOD 'immediate, urgent, and expedited'), those who could not recall their fasting time, patients undergoing re-do surgeries during the same admission, patients with documented dementia, and pregnant and diabetic patients [15].

Data analysis

Data computation and analysis were carried out with Microsoft Excel 2021 (Microsoft® Corp., Redmond, WA) and SPSS version 23 (IBM Corp., Armonk, NY, US). Standard deviations (SD) and means were used to present data that were normally distributed. To assess statistical significance, the Mann-Whitney U test was employed. Non-parametric data were reported using medians and interquartile ranges (Q1, Q3). To evaluate the statistical significance between categorical variables, chi-squared analysis was applied and p<0.05 was considered as statistically significant. The exponential of the regression coefficient (β) was computed to establish a ratio effect measure (eβ), which was subsequently used for calculating the effect measures. Confounding variables, such as age, type of surgery, and health status, were controlled for using multivariate regression models. Sensitivity analyses were performed to assess the impact of any missing data on the final results. P-values in the multivariable analysis were determined using the likelihood ratio test. In subgroup analysis, procedures were categorized based on whether they were day cases or required overnight hospitalization. Morning surgeries were scheduled between 8:30 am and 1:00 pm, while afternoon surgeries were scheduled from 1:00 pm to 6:00 pm.

Ethics and permissions

As this study constituted a clinical audit of existing service provision, formal ethical approval was not necessary. However, it was registered with the hospital's local audit office, and it adhered to information governance and auditing standards. Before commencing the study, verbal consent was obtained from participants. While written consent was not acquired, participants were actively engaged in providing information regarding preoperative fasting.

## Results

Patients’ recruitment

**Figure 1:**
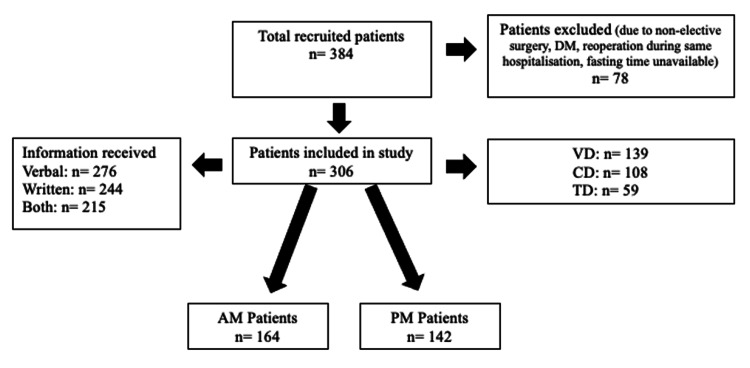
Participants’ recruitment. This figure demonstrates patients’ recruitment, inclusion and exclusion after recruitment, division into the two groups, and the form of instructions received. AM, patients with surgeries in the morning; CD, cardiac department; PM, patients with surgeries in the afternoon; TD, thoracic department; VD, vascular department

Patients were recruited consecutively from the three surgical departments based on their scheduled surgery dates, ensuring a representative sample across cardiac, thoracic, and vascular surgeries. A total of 384 patients scheduled for elective operations were recruited into the study (September-December in 2023); out of which 78 were excluded because they did not meet the inclusion criteria. Out of the remaining 306 patients included in the study, 139 (45.4%) were from the vascular surgery department (VD) and had procedures for lymphatic, arterial, and venous pathologies, 108 (35.4%) from the cardiac surgery department (CD) hospitalized for different procedures including different mitral valve procedures, and 59 (19.3%) from thoracic surgery department (TD) [16-18]. The number of patients who received both verbal and written instructions on fasting was 48 (53.3%) out of 90 patients before and 215 (70.3%) out of 306 patients after the implementation of the action plan. Of all patients, 276 (90.2%) confirmed that they had received verbal information on preoperative fasting and 244 (79.7%) had received written information, whereas 215 (70.3%) remembered getting information verbally and in writing. Every patient (306, 100.0%) was given preoperative fasting instructions in one form or another (Figure 1).

Demographic characteristics of patients

The majority of the patients scheduled for surgery were females (161 (52.6%)). More patients were scheduled for AM surgeries (164 (53.6%)) than PM surgeries (142 (46.4%)) with an average mean age of 56.6±10.3 and 58.7±9.7 years for AM and PM patients, respectively. In the majority of cases, general anesthesia was used (218 (71.2%)) compared (88 (28.8%)) to regional anesthesia, since most cases, 210 (68.6%), were open procedures, while only 96 cases (31.4%) were minimally invasive. Also, the mean body mass index of all patients was observed to be 25.6 (22.1, 28.5) kg/m^2^ (Table 1).

**Table 1:** Patients’ demographic characteristics. This table is a representation of patients’ demographic features and the types of surgical procedures they received.

Patients’ demographic data	All patients (n=306)	AM patients (n=164, 53.6%)	PM patients (n=142, 46.4%)
Age (years) - Mean±SD	60.2±12.2	56.6±10.3	58.7±9.7
Gender - n (%)			
Male	145 (47.4)	78 (25.5)	67 (21.9)
Female	161 (52.6)	83 (27.1)	78 (25.5)
Body mass index (kg/m^2^), median (Q1, Q3)	25.6 (22.1, 28.5)	24.8 (21.3, 29.6)	27.0 (20.3, 30.6)
Anesthetic type - n (%)			
General	218 (71.2)	122 (39.9)	96 (31.3)
Local/regional	88 (28.8)	39 (12.7)	49 (16.1)
Type of surgery - n (%)			
Vascular	139 (45.4)	78 (25.5)	61 (19.9)
Cardiac	108 (35.3)	59 (19.3)	49 (16.0)
Thoracic	59 (19.3)	24 (7.8)	35 (11.4)
Surgical approach - n (%)			
Mini-invasive	96 (31.4)	54 (17.6)	42 (13.7)
Open	210 (68.6)	107 (35.0)	103 (33.7)

Analysis of preoperative fasting times of the entire cohort of patients in the re-audits

The overall median fasting times (Q1, Q3) were 4.5 (2.7, 7.4) hours for clear fluids and 14.5 (12.1, 19.0) hours for solid food. Substantially prolonged fasting of >12 hours from clear fluids and solid food was recorded in 43 patients (14.1%) and 231 patients (75.5%), respectively, with fasting for >24 hours from solid meals recorded in 40 patients (13.1%) (Table 2).

**Table 2:** Distribution of all patients according to months and duration of preoperative fasting (Q1, Q3). This table depicts the number of participants for each month of the audit and how long they fasted from clear fluids and solid food. IQR, interquartile range

Fasting times	September, n (%)	October, n (%)	November, n (%)	December, n (%)	Total, n (%)	Median (IQR)
Fasting from clear fluids						
≤2 hours	4 (4.4)	19 (19.8)	18 (28.6)	24 (42.1)	65 (21.2)	-
2-4 hours	10 (11.1)	23 (24.0)	17 (27.0)	16 (28.1)	66 (21.6)	-
4-6 hours	35 (38.9)	22 (22.9)	15 (23.8)	10 (17.5)	82 (26.8)	-
6-12 hours	23 (25.6)	15 (15.6)	8 (12.7)	4 (7.0)	50 (16.3)	-
>12 hours	18 (20.0)	17 (17.7)	5 (7.9)	3 (5.3)	43 (14.1)	-
Total, n	90	96	63	57	306	4.5 (2.7, 7.4)
Fasting from solid food						
6-12 hours	8 (8.8)	16 (16.7)	21 (33.3)	30 (52.6)	75 (24.5)	-
12-24 hours	67 (74.4)	64 (66.7)	36 (57.1)	24 (42.1)	191 (62.4)	-
>24 hours	15 (16.7)	16 (16.7)	6 (9.5)	3 (5.3)	40 (13.1)	-
Total, n	90	96	63	57	306	14.5 (12.1, 19.0)

**Figure 2:**
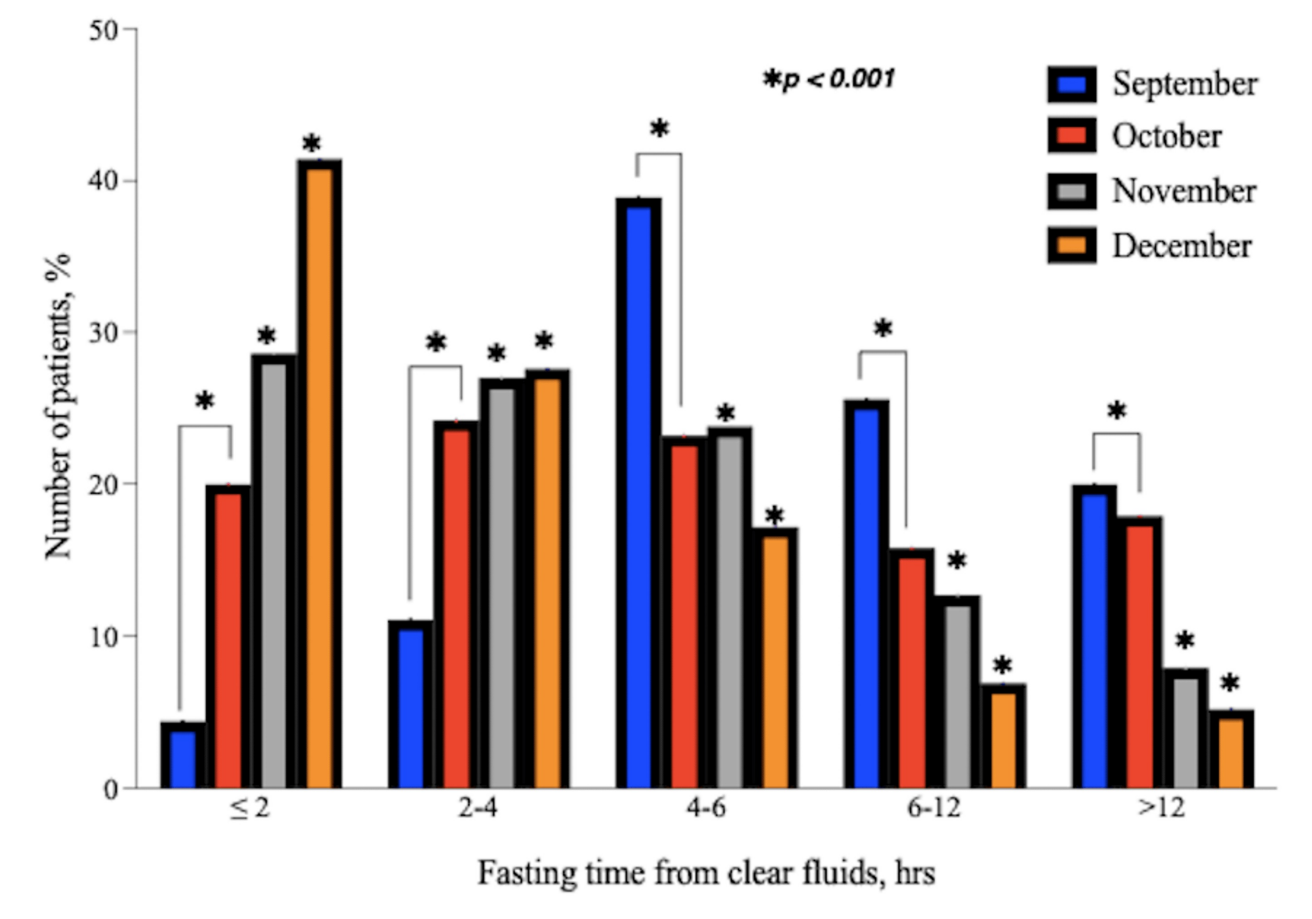
Distribution of fasting times from clear fluids according to months. These bar charts show the changes in the number of patients that fasted from clear fluids for each of the time ranges in the subsequent months following the implementation of the action plan. *p<0.001 is for monthly (October, November, and December, respectively) fasting times from clear fluids compared to the baseline (September) audit.

**Figure 3:**
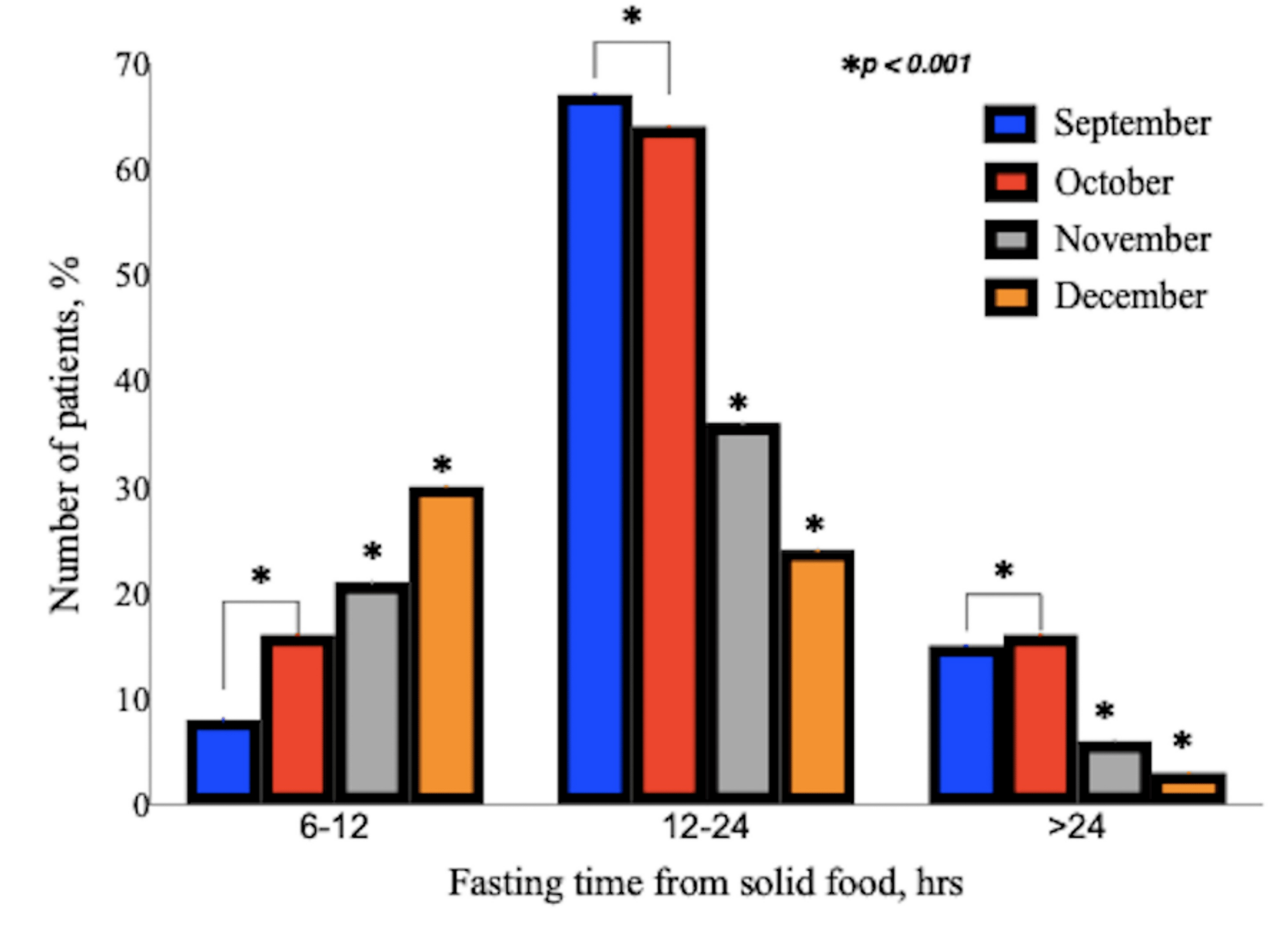
Distribution of fasting times from solid food according to months. This bar chart shows the changes in the number of patients that fasted from solid food for each of the time ranges in the subsequent months following the implementation of the action plan. *p<0.001 is for monthly (October, November, and December, respectively) fasting times from solid food compared to the baseline (September) audit.

Fasting times from both clear fluids and solid food were observed to get shorter with each re-audit; this was a significant (p<0.05) improvement compared to the baseline (September). As a result, the number of patients who fasted for ≤4 hours from clear fluids and ≤12 hours from solid food increased after each month (Figure 2 and Figure 3). Between the three departments, there were no statistically significant differences in the durations of fasting from solid food and clear fluids (p=0.091).

Comparison of fasting times between AM and PM patients

**Figure 4:**
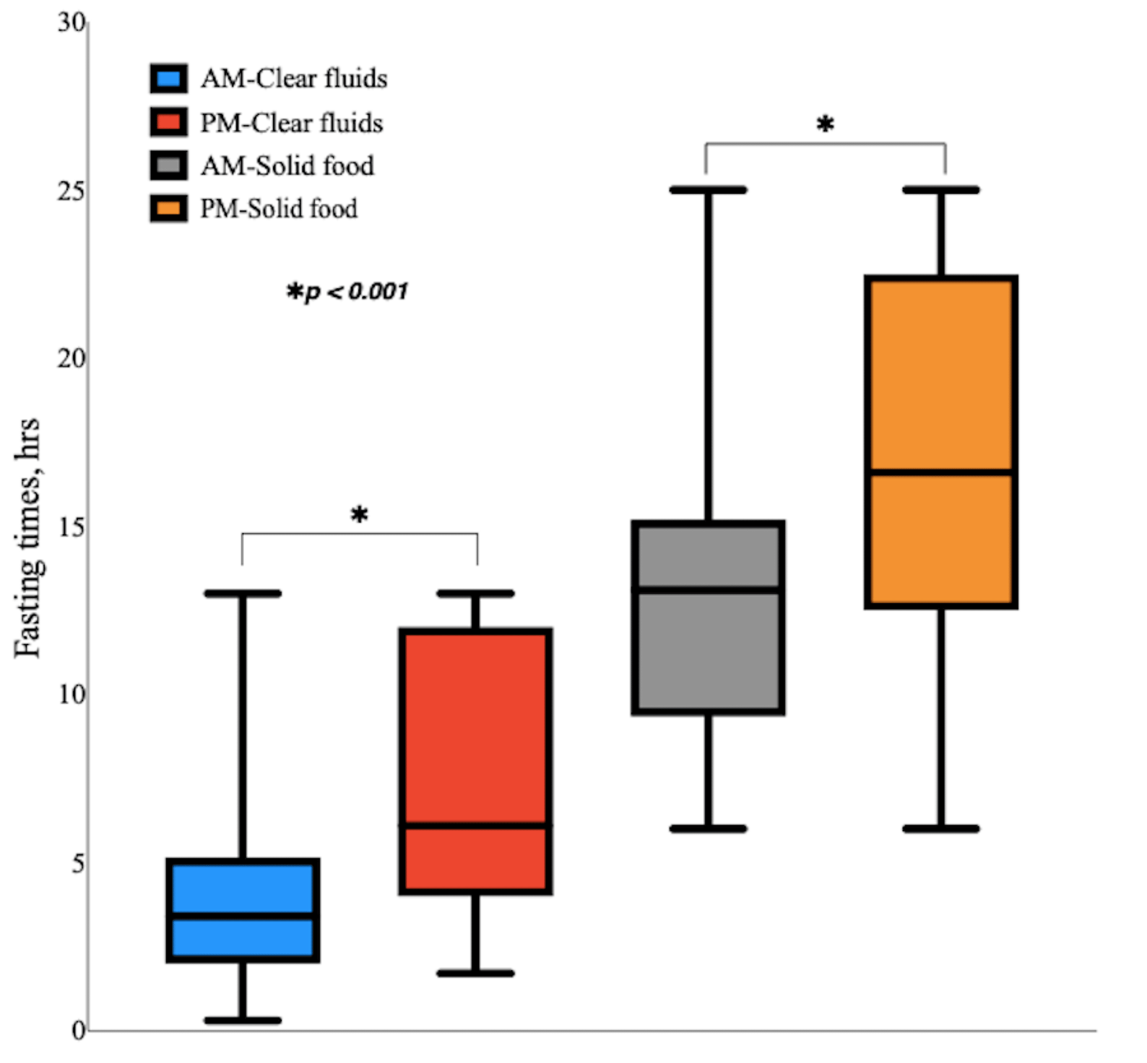
Comparison of preoperative fasting times from clear fluids and solid food in AM patients vs. PM patients. This box and whisker plot represents a comparison of the length of fasting from clear fluids and solid food in the two groups. *p<0.001 is for monthly fasting times from clear fluids and solid food between AM and PM patients

With respect to clear fluids, the majority of the AM patients (59 (36.0%)) fasted for ≤2 hours while the majority of PM patients (44 (31.0%)) fasted for six to 12 hours. For solid foods, the majority of patients in both the AM group (106 (64.6%)) and the PM group (92 (64.8%)) fasted for 12-24 hours. PM patients experienced far more prolonged fasting than AM patients. The median fasting times from clear fluids were significantly (p<0.001) higher in the PM group compared to the AM group: 6.2 (4.0, 12.0) hours vs. 3.4 (2.0, 5.2) hours, respectively. Fasting times from solid food were also significantly (p<0.001) higher in the PM group than the AM group: 16.7 (12.6, 22.6) vs. 13.2 (9.6, 15.2) hours, respectively (Figure 4).

In the multivariable analysis of preoperative fasting durations, patients scheduled for afternoon (PM) surgeries were observed to fast from clear fluids for twice the duration compared to those scheduled for morning (AM) surgeries (adjusted eβ 2.00 (1.80-2.31), p<0.001). Additionally, PM patients fasted from solid food approximately for 30% longer than AM patients (adjusted eβ 1.30 (1.20-1.41), p<0.001). Factors such as age, sex, and BMI did not demonstrate a significant influence on the length of preoperative fasting (Table 3).

**Table 3:** Unadjusted and adjusted ratios of the natural logarithm of preoperative fasting time from clear fluids and solid food. All p-values from the likelihood ratio test. A multivariable analysis of different covariates as predictors of prolonged preoperative fasting times. X(eβ), unadjusted natural logarithm of regression coefficient; Y(eβ), adjusted natural logarithm of regression coefficient

Covariates	X(e^β^), (n=306)	95% CI	P-value	Y(e^β^), (n=306)	95% CI	P-value
Clear fluids						
Surgery time (PM)	2.12	1.90-2.55	0.001	2.00	1.80-2.31	0.001
Age (years)	1.01	0.88-1.40	0.82	0.99	0.78-1.10	0.44
Sex (male)	1.01	0.88-1.40	0.82	0.99	0.78-1.10	0.44
Body mass index (kg/m^2^)	1.10	0.91-1.50	0.82	0.97	0.61-1.20	0.35
Solid food						
Surgery time (PM)	1.32	1.22-1.45	0.001	1.30	1.20-1.41	0.001
Age (years)	1.10	0.91-1.50	0.82	0.97	0.61-1.20	0.35
Sex (male)	1.01	0.88-1.40	0.77	1.00	0.78-1.10	0.44
Body mass index (kg/m^2^)	1.20	0.95-1.42	0.69	1.01	0.90-1.30	0.40

## Discussion

The outcome of this study shows poor compliance with international preoperative fasting guidelines. Patients scheduled for elective procedures in the three departments fasted for far longer than is advised with the majority of them abstaining from clear liquids for >2 hours (241 (78.8%) at the end of December) and from solid food for >6 hours (303 (99.0%) at the end of December)). Nonetheless, this showed significant improvement when compared to the fasting times among patients in the baseline audit (of 90 participants in September); 86 (95.6%) and 90 (100%) patients fasted from clear fluids for >2 hours and from solid foods for >6 hours, respectively (Table 2).

Abstinence from clear fluids and solid food was much longer among PM patients compared to AM patients: for clear fluids, 6.2 hours (4.0, 12.0) vs. 3.4 hours (2.0, 5.2), p<0.001, and for solid foods, 16.7 hours (12.6, 22.6) vs. 13.2 hours (9.6, 15.2), p<0.001. Though below the recommended level, there were significant improvements in preoperative fasting times following the implementation of the action plan. Fasting times from clear fluids for >2 hours fell from 86 (95.6%) out of 90 patients in September to 33 (57.9%) out of 57 patients in December; p=0.021. Fasting times from solid food for >6-12 hours dropped from 82 (91.1%) out of 90 patients in September to 27 (47.4%) out of 57 patients in December; p=0.005.

The results of this audit are similar to previously published reports of other studies and show nonadherence to modern perioperative recommendations [6-8]. This audit also demonstrates that many patients fast for over 12 hours before surgery, which may cause significant discomfort, thirst, and hunger. A meta-analysis showed that shorter fasting time is associated with greater postoperative comfort, lesser insulin resistance, and reduced stress response. Furthermore, the meta-analysis associated higher rates of postoperative nausea and vomiting with prolonged fasting [16].

Causes of lengthier preoperative fasting include poor staff understanding of the guidelines and challenges with efficiency and scheduling, which hinder guidelines implementation [17]. The results of this audit show that a sizable percentage of patients reported extended fasting times despite getting written information with clear instructions highlighting the importance of patient education, comprehension, and participation in decision-making. These findings can be explained by misunderstanding of preoperative instructions and reduced appetite due to preoperative stress and anxiety [18]. Poor adherence to preoperative fasting guidelines can result from unclear instructions. Initially, guidelines recommended fasting "at least" two hours rather than "no more than," possibly leading patients to believe that longer fasting periods would be beneficial [19]. Subsequently, these guidelines were revised to encourage adults to consume clear fluids up to two hours before elective surgery [19]. It is important to note that while most surgical patients receive written fasting instructions, they are often not provided with explanations regarding the reasons for these guidelines.

Some anesthesiologists still advocate for prolonged preoperative fasting to prevent aspiration during anesthesia induction. However, a study by Murphy et al. demonstrated no cases of aspiration following the adoption of the more lenient "new guidelines" [17]. The less a patient is informed and educated about preoperative fasting, the higher the probability of nonadherence. Among our patient groups, all 28 (9.2%) patients who did not get written instructions had longer mean fasting times from solid foods and clear fluids (p=0.025).

This study reveals that PM patients observed significantly longer fasting periods before elective procedures compared to AM patients (p<0.0001). This contrasts with the findings of El-Sharkawy et al., who reported no significant difference in fasting times between the two groups [20]. This discrepancy may be linked to the previously discussed factors contributing to longer-than-recommended fasting times as well as the generally longer preoperative period for PM patients compared to AM patients.

Improving compliance can be achieved by combining verbal and written instructions with clear explanations during elective surgeries, rather than relying solely on written advice [21]. Giving patients written instructions was linked to increased patient understanding, higher levels of satisfaction with care, and lower rates of readmission, as evidenced by earlier researches of a similar nature [22]. Comparably, research by Segador et al. showed that, in comparison to verbal instructions alone, written instructions along with verbal instructions greatly boosted compliance with medications [23].

Newton et al. demonstrated a significant improvement in adherence to preoperative fasting guidelines after implementing a systematic audit process and making substantial changes [24]. They introduced interventions that included an opt-out system, allowing anesthetists and surgeons to exempt patients from strict fasting guidelines when longer fasting periods were deemed necessary. Other institutions have also adopted effective strategies, such as the "Think Drink" initiative, which provides staff with algorithms to improve coordination and communication across theaters, wards, and admission areas. These initiatives have proven successful in enhancing adherence to fasting recommendations [25].

There are numerous clinical situations when extended preoperative fasting may be necessary. Clinical judgment is still crucial, particularly for patients who have a high risk of aspiration, including those who are scheduled for emergency procedures or have health issues that could slow the transition of food from the stomach [26]. When there is a likelihood of impromptu general anesthesia, as it does happen in emergency surgical patients, over-fasting may be justified; nonetheless, written and verbal instructions will be beneficial in such cases [27].

Preoperative carbohydrate loading, administered the night before and two hours prior to surgery, has been proposed as a beneficial strategy to reduce fasting durations and mitigate insulin resistance development. However, this approach is not currently implemented in our institution. Despite ongoing discussions regarding the clinical advantages of these carbohydrate drinks in reducing postoperative complications, a systematic review and meta-analysis indicated that high-carbohydrate drinks did not significantly affect surgical outcomes [12]. Despite this uncertainty, Enhanced Recovery After Surgery (ERAS) programs, aimed at enhancing recovery and minimizing the catabolic effects of surgery, now incorporate carbohydrate loading protocols [19].

Limitations

This audit has several strengths. First, it was conducted in different departments using patients who underwent different surgical procedures. Furthermore, by including patients who had operations at different times (AM and PM), it is possible to compare various preoperative surgical pathways and how they affect patient optimization prior to surgery. Furthermore, it was ensured that the validity and accuracy of the data presented were not unduly skewed by excluding diabetic patients, elective surgeries that turned "emergent" due to the patients' worsening conditions, and reoperations within a single hospitalization when fasting rules are not applicable. However, there is a potential for selection bias given that this study is a single-center quality improvement initiative, and intra-hospital guidelines concerning outcome data could influence results, limiting their generalizability to the broader surgical patient population. Despite the prospective data collection approach, reliance on patient recall might introduce additional bias. This study also did not compare the postoperative outcomes between patients adhering to current international fasting guidelines and those fasting for longer periods, warranting further investigation into the impact of preoperative fasting duration on postoperative outcomes. Based on the outcome of this study, a continuous re-audit was recommended in order to gradually improve the compliance rate to accepted levels.

## Conclusions

This study confirmed prolonged preoperative fasting among elective surgical patients and revealed for the first time (to the best of our knowledge) that PM patients fasted for longer periods than AM patients. It also showed that the implementation of clinical recommendations is slow. Prolonged preoperative fasting is the result of several different factors, with poor conveyance of fasting instructions to patients, patients’ misinterpretation of such information, and patients’ personal preference for fasting as the major contributing factors. Ensuring adherence to standardized fasting protocols requires training staff, ensuring effective clinical communication, and delivering clear written and verbal instructions to every surgical patient.

## Appendices

**Table 4:** Standardized questionnaire to determine preoperative length of fasting.

PLEASE ANSWER THE FOLLOWING QUESTIONS AS BEST AS YOU CAN REMEMBER	
QUESTION	ANSWER
1. How many hours (or minutes) ago did you have your last drink?	
2. How many hours (or minutes) ago did you have your last meal (solid food)?	
3. How did you learn when to stop eating and drinking before your operation? Circle YES or NO.	
I saw it in my preoperative to-do list only	YES or NO
I was told at the preoperative clinic only	YES or NO
I saw it in my preoperative to-do list and was told at the pre-operative clinic	YES or NO
